# Mechanical Stimulation Protects Against Chondrocyte Pyroptosis Through Irisin-Induced Suppression of PI3K/Akt/NF-κB Signal Pathway in Osteoarthritis

**DOI:** 10.3389/fcell.2022.797855

**Published:** 2022-03-09

**Authors:** Shuangshuo Jia, Yue Yang, Yishu Bai, Yingliang Wei, He Zhang, Yicheng Tian, Jiabao Liu, Lunhao Bai

**Affiliations:** ^1^ Department of Orthopedics, Shengjing Hospital of China Medical University, Shenyang, China; ^2^ Jiangsu Hengrui Pharmaceuticals Co., Ltd., Shanghai, China

**Keywords:** irisin, osteoarthritis, exercise, PI3K/Akt/NF-κB, pyroptosis, chondrocyte

## Abstract

Irisin, a myokine secreted by muscle during physical exercise, is known to have biological activities in different cell types. Chondrocyte inflammation and pyroptosis have been shown to play important roles in osteoarthritis (OA). In this study, we investigated the effects of exercise-induced irisin during different intensities of treadmill exercise in a rat OA model and the anti-inflammatory and antipyroptosis mechanism of irisin in OA chondrocytes. Forty-eight SD rats (*n* = 8) were randomly assigned to control (CG), OA (OAG), OA groups under different intensities of treadmill exercise (OAL, OAM, and OAH), OAM + irisin neutralizing antibodies group (OAM + irisin (NA)). The levels of irisin and the severity of OA between groups were detected using ELISA, histology, immunohistochemistry, X-ray and computed tomography and magnetic resonance imaging. The anti-inflammatory and antipyroptosis mechanisms of irisin were investigated *in vitro* in OA chondrocytes preincubated with recombinant irisin (0, 5, or 10 ng/ml) for 1 h before treatment with interleukin-1β (IL-1β) for 24 h mRNA and protein expression levels were determined using quantitative reverse transcription polymerase chain reaction, and western blot analyses. Morphological changes and cell death associated with pyroptosis were examined using transmission electron microscopy, flow cytometry and immunofluorescence. Moderate-intensity treadmill exercise increased the levels of irisin, exhibiting the best therapeutic effects on OA which could be suppressed by irisin neutralizing antibodies. Irisin not only recovered the expression of collagen II and attenuated that of MMP-13 and ADAMTS-5 in IL-1β-induced OA chondrocytes by inhibiting the PI3K/Akt/NF-κB signaling pathway, but also inhibited the activity of nod-like receptor protein-3 (NLRP3)/caspase-1, thus ameliorating pyroptosis in chondrocytes. In conclusion, moderate mechanical stimulation protects against chondrocyte pyroptosis through irisin-induced suppression of PI3K/Akt/NF-κB signal pathway in osteoarthritis.

## 1 Introduction

Osteoarthritis (OA) is one of the most common joint diseases, affecting more than 80% of the elderly over the age of 70 (Martel-Pelletier et al., 2016). OA is often accompanied by inflammation of articular cartilage, degeneration, and synovitis (Kalunian, 2016). Current treatments for OA include surgery and drug therapy such as nonsteroidal anti-inflammatory drugs (NSAIDs); however, these only relieve symptoms. Nonsurgical therapies such as physical exercise are recommended as first-line treatment in the new clinical guidelines (Block, 2014; Nelson et al., 2014), with exercise therapy being widely accepted as a safe and effective treatment. Over the past 10 years there has been increasing evidence that physical activity has a positive effect on OA (Fransen et al., 2014; Fransen et al., 2015). However, the mechanisms by which physical exercise improves OA and achieves a therapeutic effect remains unclear.

Boström et al. (2012) found in 2012 that physical exercise stimulates muscles to produce the metabolic factor irisin, a hormone-like polypeptide cleaved by fibronectin type III domain-containing protein 5 (FNDC5). Irisin contains 112 amino acid residues, having a molecular weight of approximately 12 kD. Studies have shown that exercise could increase the expression of peroxisome proliferator-activated receptor-γ (PPAR-γ) and its coactivator-1-α (PGC-1α) in muscles, thus facilitating the downstream generation of FNDC5 and its proteolytic cleavage to form irisin (Hecksteden et al., 2013; Aydin, 2014; Norheim et al., 2014). Although irisin has been reported to exert anti-osteoarthritic effects in chondrocytes (Li et al., 2021; Vadala et al., 2020), its role in the exercise therapy of osteoarthritis (OA) remains unknown. As an exercise-induced myokine, we hypothesized that irisin could be a key exercise factor in the treatment of OA.

Even though the etiology and pathogenesis of OA remain unknown, inflammatory responses in chondrocytes, which trigger and promote chondrocyte death, have been known to play critical role in the development of OA (Scanzello, 2017a; 2017b). Pyroptosis is a type of proinflammatory programmed cell death, which is triggered by inflammation involving the activation of nod-like receptor protein-3 (NLRP3)/caspase -1 (Y. Hu et al., 2020; Kesavardhana et al., 2020). Improving the understanding of inflammation and inflammation-induced pyroptosis in OA could aid in finding novel therapeutic targets.

To identify the exercise-related differentially expressed genes, we used data obtained from the Gene Expression Ominibus database (GSE74898), gene ontology (GO) annotation and Kyoto Encyclopedia of Genes and Genomes (KEGG) for our analyses. Several studies have shown that the nuclear factor kappa B (NF-κB) transcription factor plays a central role in the pathogenesis of osteoarthritis (Choi et al., 2019; Lepetsos et al., 2019; Chow & Chin, 2020). Based on the previous studies and the exercise-related differentially expressed genes, we hypothesized that the nuclear factor kappa B (NF-κB) pathway plays a key role in exercise therapy for OA (Supplementary Figures S1–S3). PI3K/Akt, which is upstream of NF-κB and belongs to the serine/threonine protein kinases family, is involved in the activation of the NF-κB pathway through its phosphorylation (Z. C. Hu et al., 2019; Lou et al., 2017). The activated PI3K/Akt/NF-κB cascade was reported to increase the expression of matrix metalloproteinases (MMPs) and that of a disintegrin and metalloproteinase with thrombospondin motifs (ADAMTS), whereas decrease that of collagen II, eventually leading to the destruction of the extracellular matrix (ECM) and inducing chondrocyte pyroptosis during the pathogenesis of OA (Huang et al., 2019; Xie et al., 2019). Thus, it is important to investigate the PI3K/Akt/NF-κB pathway and the mechanism of pyroptosis as potential targets for OA therapy.

In the present study, we evaluated the levels of irisin in human articular cartilage and rats following treadmill exercise compared with those in unexercised control animals, and also investigated the therapeutic effects of irisin on OA during treadmill exercise. We also investigated the mechanism by which irisin affects the PI3K/Akt/NF-κB signaling pathway to inhibit inflammation and the antipyroptosis mechanism in chondrocytes.

## 2 Materials and Methods

### 2.1 Bioinformatics Analysis

To identify the exercise-related differentially expressed genes, we selected the GSE74898 dataset for further study. This dataset was obtained from the GEO database (https://www.ncbi.nlm.nih.gov/geo) and contained Sprague-Dawley (SD) rats divided in a control sedentary group and 2-, 5-, or 15-d exercise groups. All groups were used for differential gene expression analysis. Volcano plots of differentially expressed genes were generated using the Limma/R package (version 3.5.1). The primary parameters was performed as log fold change and a log fold change >2 indicated significantly differentially expressed genes. Then, differentially expressed genes were submitted to the DAVID v6.8 tools (https://david.ncifcrf.gov/) for annotation, visualization, and integrated discovery. Gene ontology (GO) functional annotation analysis (Molecular function, Biological process, and Cellular component) was adopted on the exercise-related differentially expressed genes and Kyoto Encyclopedia of Genes and Genomes (KEGG) pathway enrichment analysis was used to identify the potential pathways in exercise therapy (Huang da et al., 2009).

### 2.2 Human Articular Cartilage and Clinico-Pathological Characteristics

The protocol and experiments on joint specimen after human total knee arthroplasty were approved by the Ethics Committee of Shengjing Hospital of China Medical University (No: 2019PS629K) which abides the principles set out in the World Medical Association Helsinki Declaration. Twelve donors participated in our study and we obtained informed consent from all of our patients. As mentioned in our previous study (Lin et al., 2021), the cartilage and the clinicopathological features of patients with knee osteoarthritis after total knee replacement were collected and divided into damaged and undamaged groups according to the International Cartilage Repair Society (ICRS) grade (Kleemann et al., 2005), ICRS = 0 was classified as the undamaged region, and the ICRS = 1 ∼ 4 was classified as the damaged region. We compared the damaged area with the undamaged area in the same donors.

### 2.3 Experimental Animals and Osteoarthritis Model

A total of 48 Sprague-Dawley (SD) rats (240 ± 5 g, 4 weeks of age, male, and specific-pathogen-free, eight rats per group, and six rats per cage) were obtained from HFK Bioscience Co. Ltd. (Beijing, China). The present study was approved by the Ethics Committee of China Medical University (no. 2017PS237K) which abides the principles set out in the World Medical Association Helsinki Declaration. All SD rats were placed in separate cages covered with sawdust and kept in a controlled environment (12-h light:12-h dark cycles, temperature controlled at 22 ± 2°C with 70% humidity), with food and water ad libitum. The weight of rats was recorded at fixed periods. After adaptive feeding for 1 week, all rats started adaptive training in the ZH-PT animal treadmill exercise platform (Zhongshidichuang Science & Technology Development Co. Ltd., Beijing, China). The rats were anesthetized with pentobarbital sodium (1.0%, 2.5 ml/100 g), then MIA (1 mg per cavity in 50 μl sterile saline) was intra-articular injected by micro syringe to establish the OA models.

### 2.4 Treadmill Exercise Protocols With Osteoarthritis Rat Models

**FIGURE 1:**
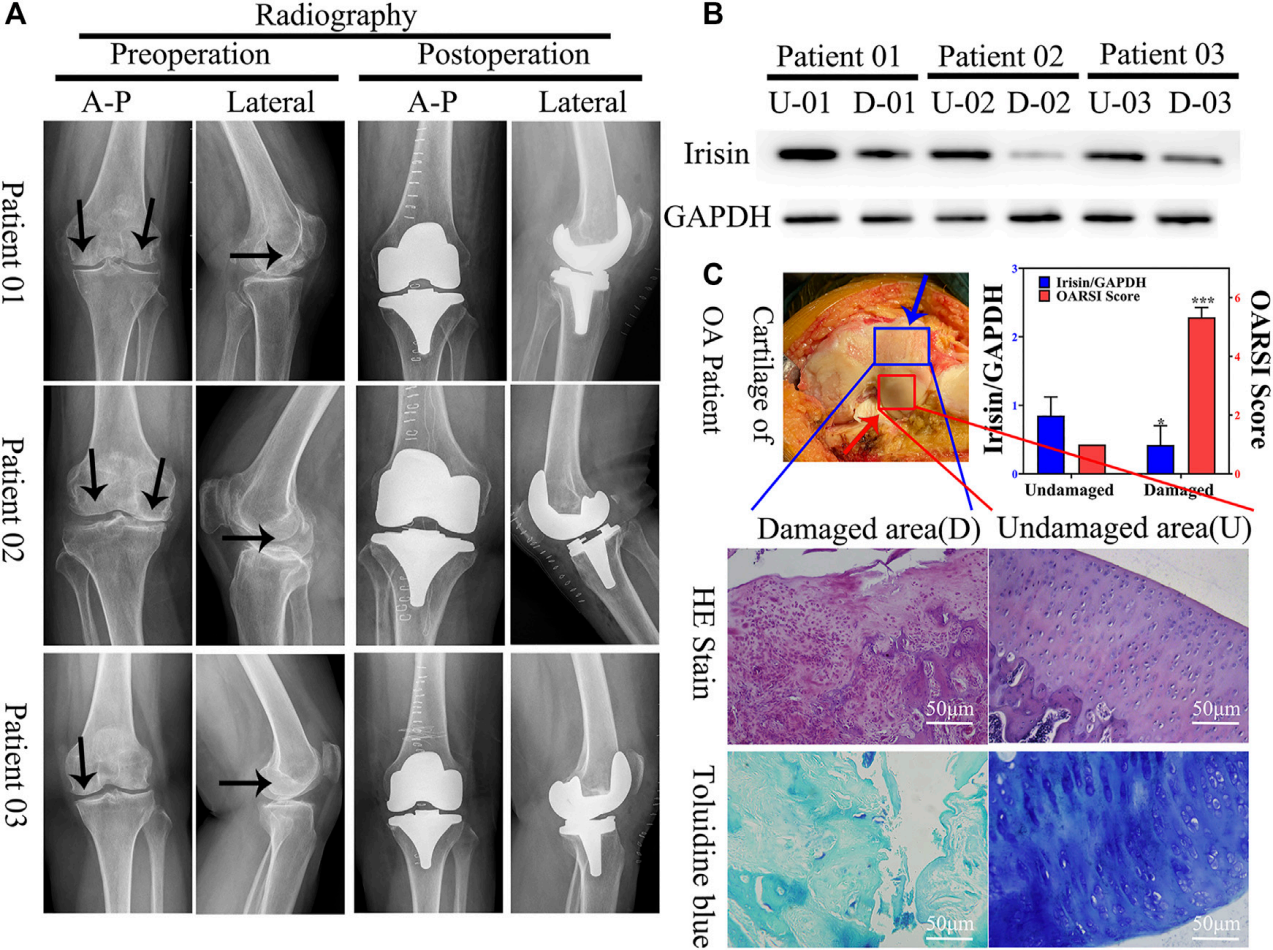
Expression of irisin is downregulated in the damaged area of the articular cartilage in patients with knee osteoarthritis (OA). **(A)** Plain radiograph of patients with knee osteoarthritis (OA) (*n* = 12). Black arrow indicate areas with more severe osteoarthritis. Anterior–posterior (A-P). **(B)** Relative protein level of irisin in undamaged (U) and damaged **(D)** areas, with GAPDH as control. **(C)** H&E and toluidine blue staining of knee articular cartilage. Blue arrow indicate damaged area, red arrow indicate undamaged area. Relative level of irisin and OARSI score. *p < 0.05; **p < 0.01; ***p < 0.001.

The SD rats were trained on the treadmill at 10 m/min and 10 min/d for 1 week without any other stimulation to adapt to the treadmill training. The treadmill exercise protocols used in this study were based on previous studies (Yang et al., 2020; Yang et al., 2018). The specific treadmill exercise protocols are shown in Figure 1A. The control group (CG) and osteoarthritis (OAG) were the sedentary groups, whereas the OA with low-intensity treadmill exercise (OAL); OA with moderate-intensity treadmill exercise (OAM); OA with high-intensity treadmill exercise (OAH) were the exercise groups. All tests were performed using appropriate light, acoustic and electrical stimulations. To provide data on the importance of irisin in exercise therapy of osteoarthritis, irisin neutralizing antibodies were added *in vivo* to the moderate-intensity exercise group. The rats were injected with 20 μg irisin neutralizing antibody *via* the tail vein 1 h before exercise, three times a week according to the previous study (Fan et al., 2019; Li et al., 2017).

### 2.5 Tissue Sampling and Collection

Rats were sacrificed *via* pentobarbital overdose after the last treadmill exercise session. Blood was collected immediately from the inferior vena cava and centrifuged at 3,000 rpm for 15 min to separate the serum. The supernatant was removed, transferred to microtubes and stored at −80°C for analysis. Rats were dissected to obtain the knee joints which were then fixed with 4% paraformaldehyde (Sigma-Aldrich, St. Louis, MO, United States) solution for 7 d. The knee joints were then decalcified using 20% EDTA (Sigma-Aldrich) solution for 7 weeks which was replaced every 3 days with fresh solution. After decalcification, the joints were dehydrated in ethanol and xylene (Sigma-Aldrich). Finally, the samples were embedded in paraffin (Sigma-Aldrich).

### 2.6 Enzyme-Linked Immunosorbent Assay of Levels of Irisin

The serum levels of irisin and those of the synovial fluid (SF) irisin were determined using a commercial enzyme-linked immunosorbent assay (ELISA) kit (SEN576Ra; Cloud-Clone Corp. USCN Life Science, Wuhan, China) on a spectrophotometric reader at a wavelength of 450 nm, following the manufacturer’s instructions. The standard curve *R* ^2^ = 0.997.

### 2.7 X-Ray and Computed Tomography and Magnetic Resonance Imaging Observation

Knee joint images were captured by X-ray (MX-20, Faxitron X-ray, Corp., Lincolnshire, IL, United States) and SkyScan 1276 Micro-CT (Bruker, Kontich, Belgium) and NRecon version 1.6 software (Bruker) and Ingenia3.0 T MRI system (Philips, United States). The rats were anesthetized by intraperitoneal injection of pentobarbital sodium (40 mg/kg) and fixed in supine position. The bilateral ankles were fixed on the tray with adhesive tape. The lens was focused at an appropriate focal length on the knee joint of the rat and the exposure time was set to apropriate minutes to ensure a clear image. The extent of osteoarthritis was assessed by imaging findings, including joint space narrowing and articular surface calcification, as well as articular cartilage damage, according to the imaging scoring system used in previous literature with macroscopic score which was based on surface roughness and erosin. Using imaging techniques, we quantified the tibial plateau or femoral condyle surface by calculating the ratio of the lesion area to the total surface area. Both the tibial and femoral joints were evaluated based on a maximum score of 10 (Gerwin et al., 2010; Kohn et al., 2016; Lin et al., 2021). The scoring was performed by two experienced observers who were blinded to the study groups.

### 2.8 Histology

After the samples were embedded in paraffin, 5-μm sagittal sections were cut from the tibiofemoral joints and stained with hematoxylin and eosin (H&E) and toluidine blue for histological examination. Obtained sections were then visualized under an Olympus BX53 microscope (Olympus, Tokyo, Japan). The degree of articular cartilage damage in the tibiofemoral joint was assessed based on the Modified Mankin scale (0–14 points) and the Osteoarthritis Research Society International (OARSI) scale (0–24 points) (Gerwin et al., 2010). Both the tibial and femoral joints were evaluated based on a maximum Mankin score of 28 and OARSI score of 48. The scoring was performed by two experienced observers who were blinded to the study groups.

### 2.9 Immunohistochemistry

After sections were deparaffinized and washed with PBS, the following steps were performed under humidifying conditions. Enzymatic antigen retrieval (C1033, Solarbio Science & Technology Co., Ltd., Beijing, China) was performed at 37°C for 30 min to repair antigens in the sections. Then, 3% H_2_O_2_ was used at 25°C for 30 min to eliminate endogenous peroxidase activity, while blocking serum (15019, Cell Signaling Technology, Danvers, MA, United States) was used at 25°C for 30 min to block nonspecific antigens. Then, sections were incubated with anti-FNDC5 antibody (ab174833, 1:100, Abcam, Cambridge, MA, United States), anti-collagen II antibody (ab34712, 1:100, Abcam), anti-MMP-13 antibody (ab39012, 1:100, Abcam), anti-ADAMTS-5 antibody (ab41037, 1:100, Abcam), anti-NF-κB p65 antibody (ab16502, 1:100, Abcam), anti-NLRP3 antibody (19771-1-AP, 1:100, Proteintech Group, Rosemont, IL, United States), and anti-caspase 1 antibody (22915-1-AP, 1:100, Proteintech Group) at 4°C overnight. The next day, a Boost IHC Detection Reagent (HRP, rabbit; 8114, Cell Signaling Technology) was added to sections at 25°C for 30 min and then sections were washed thrice with PBS. Sections were stained using a diaminobenzidine (DAB) substrate kit (8059, Cell Signaling Technology) for 3 min and counterstained with hematoxylin (G8550, Solarbio Science & Technology Co., Ltd., Beijing, China) for 3 min. Then, sections were dehydrated and sealed. Images of sections were captured using optical microscopy (Eclipse Ci, Nikon, Chiyoda, Japan) and optical density was quantified using the Image-Pro Plus version 6.0 software. The average optical density represented the relative expression of collagen II, while the expression of MMP-13, ADAMTS-5, irisin, NLRP3 and caspase-1 was measured based on the percentage of positively-stained cells.

### 2.10 Primary Chondrocyte Culture and Treatment

Rat primary chondrocytes were isolated from the knee articular cartilage of 4-week-old SD rats. Cartilage from the knee joints was cut into 1 mm^3^ particles. Then, 3 mg/ml pronase K (V900887, Sigma-Aldrich, St. Louis, MO, United States) was used to digest the cartilage fragments for 2 h at 37°C, followed by digestion with 2.0 mg/ml collagenase D (C0130, Sigma-Aldrich) for 1 h at 37°C. Mild mechanical vibrations were used throughout the digestion process. Next, cell suspensions were centrifuged at 800 rpm for 5 min and resuspended in fresh Dulbecco’s modified Eagle’s medium F-12 (DMEM/F-12; Thermo Fisher Scientific, Carlsbad, CA, United States). Resuspended chondrocytes were transferred into culture flasks and cultured with DMEM/F-12 supplemented with 10% FBS (abs972, Absin Bioscience Inc., Shanghai, China) and 1% penicillin with streptomycin (Hyclone Laboratories Inc., Logan, UT, United States) at 37°C in a 5% CO_2_ incubator. Then, the chondrocytes were passaged at a 1:4 ratio with 0.05% trypsin (C0202; Beyotime Biotech, Shanghai, China) until 70%–80% confluence. Chondrocytes were cultured between 1 and 3 passages to avoid loss of the chondrocyte phenotype. According to the previous study (Tine Kartinah et al., 2018; Shirvani et al., 2019), chondrocytes were pretreated with irisin (8880-IR-025, R&D Systems, Minneapolis, MN, United States) dissolved in culture media at different physiological concentrations (0, 5, or 10 ng/ml) for 1 h based on the synovial fluid irisin concentrations in rats and then incubated with or without IL-1β (10 ng/ml) for 24 h to induce chondrocyte inflammation and the involvement of signaling pathways. Chondrocytes were divided into the following groups: control group, in which the same amount of serum-free medium was added; IL-1β group; IL-1β + irisin (5 ng/ml) group; IL-1β + irisin (10 ng/ml) group.

### 2.11 Cell Viability Assay

The optimum applied concentration of IL-1β in primary rat chondrocytes was evaluated using the CCK8 assay (Beyotime Biotech, Shanghai, China). Chondrocytes (1 × 10^4^/well) were seeded onto a 96-well culture plate and treated with incremental concentrations of IL-1β (1, 2, 4, 8, 10, 15, and 20 ng/ml). The concentrations of IL-1β used in subsequent experiments were selected based upon these results. Measurement of optical densities values at 450 nm was performed using a Gen5 plate reader (BioTek, Winooski, VT, United States). All experiments were carried out in triplicate.

### 2.12 Western Blot Analysis

Human cartilage was washed in cold PBS three times, then cut into pieces. Cartilage and rat primary chondrocytes were both lysed in RIPA (9806S, Cell Signaling Technology) with 1 mM PMSF (ST506; Beyotime Biotech, Shanghai, China) and 1 mM phosphatase inhibitors (P1081; Beyotime Biotech, Shanghai, China). The lysates were centrifuged at 12,000 rpm/min for 15 min at 4°C and the supernatants were collected and stored at −80°C. Protein concentration was measured using the bicinchoninic acid assay (P0010; Beyotime Biotech, Shanghai, China). Equal amount of proteins (20 μg) was separated using polyacrylamide gel electrophoresis (8%–10% SDS-PAGE) and then transferred onto polyvinylidene difluoride (PVDF) membranes. Next, PVDF membranes were blocked with 5% bovine serum albumin (BSA) for 2 h at 25°C and washed thrice using Tris-buffered saline (TBS) with 0.1% Tween-20 (TBST). PVDF membranes were then incubated with primary antibodies at 4°C overnight. Antibodies used included anti-FNDC5 antibody (ab174833, 1:1,500, Abcam), anti-collagen II antibody (ab188570, 1:2,000, Abcam), anti-MMP-13 antibody (ab39012, 1:2,000, Abcam), anti-ADAMTS-5 antibody (ab41037, 1:2,000, Abcam), anti-PI3K antibody (ab32089, 1:2,000, Abcam), phosphorylated (p)-PI3 kinase antibody (4228T, 1:2,000, Cell Signaling Technology), anti-Akt antibody (abs131788, 1:2,000, Absin Bioscience Inc.), phosphorylated (p)-Akt antibody (4058L, 1:2,000, Cell Signaling Technology), anti- NF-kappaB p65 antibody (ab16502, 1:2,000, Abcam), phosphorylated (p)-NF-kappaB p65 antibody (S536, 1:2,000, Cell Signaling Technology), NLRP3 polyclonal antibody (19771-1-AP, 1:2,000, Proteintech Group, Rosemont, IL, United States), caspase 1/p20/p10 polyclonal antibody (22915-1-AP, 1:2,000, Proteintech Group), and anti-β-actin antibody (205361-AP, 1:3,000, Proteintech Group). The next day, PVDF membranes were washed thrice with TBST and then incubated with goat anti-rabbit IgG H&L (HRP) (ab6721, 1:10,000, Abcam) for 2 h at 25°C. Membranes were visualized using enhanced chemiluminescence (Millipore, Billerica, MA, United States). ImageJ software (imagej.nih.gov/ij/download) was used for quantification. β-actin was used as internal control.

### 2.13 Quantitative Reverse Transcription Polymerase Chain Reaction Analysis

Total RNA was extracted from primary rat chondrocytes using the RNAiso Plus kit (Vazyme Biotech Co., Ltd., Nanjing, China). Next, RNA was reverse transcribed into cDNA using the HiScript II Q RT SuperMix for qPCR (+gDNA wiper; Vazyme Biotech Co., Ltd.) following the manufacturer’s instructions. The qRT-PCR reaction was prepared using a SYBR Green PCR kit (Vazyme Biotech Co., Ltd.) with the Applied Biosystems 7500 Real-Time PCR System. Each reaction was performed in triplicate. PCR conditions were as follows: step 1, 95°C for 30 s; step 2, 95°C for 5 s, and 40 cycles at 60°C for 30 s; step 3, 95°C for 15 s, 60°C for 60 s, and 95°C for 15 s. Relative mRNA expression was calculated using the 2^−ΔΔCq^ method (Livak & Schmittgen, 2001). Obtained values were represented based on the fold-change relative to β-actin. The target gene primers were designed by and purchased from Sangon, China (Table 1). β-actin was used as internal control.

**TABLE 1:** Sequences of primers used for qRT-PCR.

Target gene	Forward primer 5′-3′	Reverse primer 3′-5′
MMP-13	ATA​CGA​GCA​TCC​ATC​CCG​AGA​CC	AAC​CGC​AGC​ACT​GAG​CCT​TTT​C
ADAMTS-5	TCC​TCT​TGG​TGG​CTG​ACT​CTT​CC	TGG​TTC​TCG​ATG​CTT​GCA​TGA​CTG
COL2α1	GGA​GCA​GCA​AGA​GCA​AGG​AGA​AG	GGA​GCC​CTC​AGT​GGA​CAG​TAG​AC

### 2.14 Immunofluorescence Microscopy

Chondrocytes were seeded onto coverslips in a 24-well plate. Once 70% confluency was reached, cells were pretreated with irisin dissolved in culture media for 1 h and then incubated with or without IL-1β (10 ng/ml) for 24 h. Coverslips containing chondrocytes were fixed with 4% paraformaldehyde for 20 min and then permeabilized with 0.5% Triton X-100 (Solarbio Science & Technology Co., Ltd., Beijing, China) for 20 min at 25°C. Next, coverslips were blocked with 5% BSA for 30 min without washing, and then incubated with anti-NF-kappaB p65 antibody (ab16502, 1:500, Abcam) at 4°C overnight. After washing thrice in PBS, cells were incubated with anti-rabbit IgG (H + L) F (ab′)2 fragment (Alexa Fluor^®^ 488 conjugate) (4412s, 1:200, Cell Signaling Technology) for 3 h at 25°C. Nuclei were stained with 4,6-diamidino-2-phenylindole (DAPI) for 5 min. Coverslips were visualized under a confocal microscope (Olympus).

### 2.15 Flow Cytometry

Inflammatory chondrocyte death was assessed using the caspase-1 fluorescent inhibitor probe FLICA 660-YVAD-FMK (BD Biosciences, San Jose, CA, United States). After removing the media and washing 5 times, cells with membrane pores were marked with propidium iodide (PI) (BD Biosciences) and measured using a FACSCalibur flow cytometer (BD Biosciences). The doubling rate of cell mortality was normalized with that of the control group, and data were analyzed using the FlowJo software (BD Biosciences).

### 2.16 Transmission Electron Microscopy

Cells were fixed in 2.5% glutaraldehyde for 2–4 h at 25°C. Following centrifugation at low speed (800 rpm, 5 min), clumps of cells the size of mung beans could be seen at the bottom of the tube. Cell clusters were painted with 1% agarose and rinsed thrice with 0.1 M phosphoric acid buffer PB (pH 7.4) for 15 min each. Then, 1% OSO4 was added to gently lift and suspend the cell clusters. After dehydration, samples were embedded in resin. Following uranium-lead double staining (2% uranium acetate saturated alcohol solution, lead citrate; staining for 15 min each), sections were dried overnight at 25°C. Cell morphology and subcellular structures were observed using a Hitachi 800 TEM (Tokyo, Japan).

### 2.17 Statistical Analysis

All experiments were performed independently at least three times. Results are expressed as means ± standard error of the mean (SEM) using GraphPad Prism 5. Statistical analyses were performed using IBM SPSS Statistics 25.0. A *p*-value < 0.05 was considered statistically significant.

## 3 Results

### 3.1 Bioinformatics Analysis of Exercise-Related Differentially Expressed Genes and Potential Signaling Pathway

The volcano plots of the identified exercise-related differentially expressed genes between the control and 2, 5, and 15-d exercise groups are shown in Supplementary Figures S1A–C. We identified a total of 389 overlapping differentially expressed genes; our results are shown in Supplementary Figure S1D. We performed GO functional annotation analysis on the potential differentially expressed genes, including molecular function (Supplementary Figure S2A), biological process (Supplementary Figure S2B), and cellular component (Supplementary Figure S2C). We also performed KEGG pathway enrichment analysis to further investigate the potential pathways of these differentially expressed genes. As shown in Supplementary Figure S3, the NF-κB signaling pathway was included in the enriched KEGG pathways analysis. Based on the previous studies and the exercise-related differentially expressed genes, we predicted that the NF-κB pathway and its upstream effectors might play a key role in exercise therapy (Supplementary Figures S1–S3).

### 3.2 Expression of Irisin Was Downregulated in the Damaged Area of the Articular Cartilage in Patients With Knee Osteoarthritis and Related to Pathological Features

To evaluate the potential role of irisin in OA, we first verified the validity of articular cartilage specimens by collecting clinical images of 18 patients with knee OA (Figure 1A); these were divided into undamaged (U) and damaged (D) zones. Combined with western blot analyses (Figure 1B) and histological evaluation (Figure 1C), we observed that irisin was significantly (*p* = 0.046 < 0.05) downregulated in the damaged area of the articular cartilage.

### 3.3 Effects of Treadmill Exercise on the Concentration of Irisin in Osteoarthritis Rats

**FIGURE 2:**
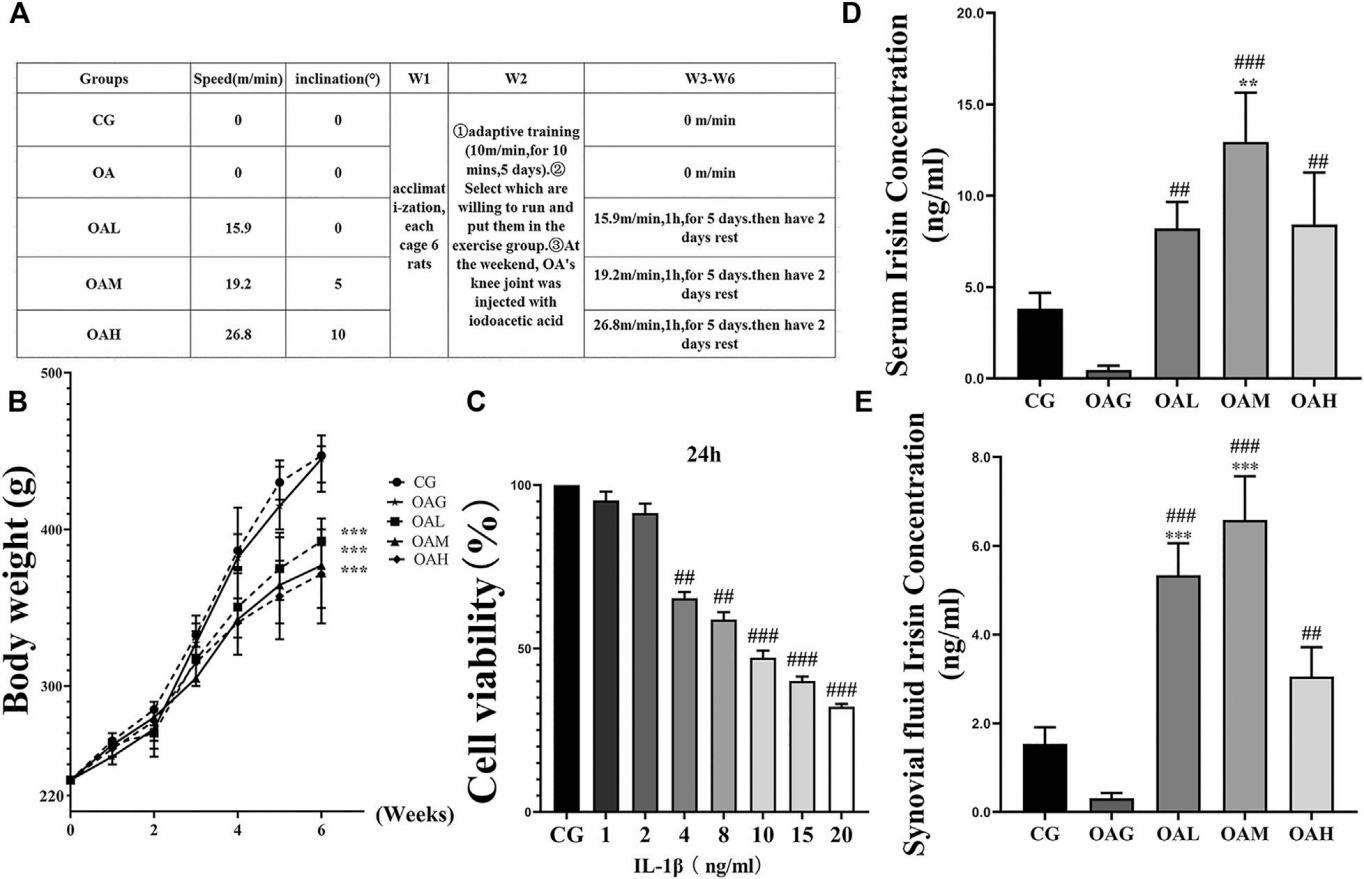
Treadmill exercise protocols established for the OA rat model and ELISA of serum and synovial fluid irisin concentrations in rats during the study period. **(A)** The different intensities of treadmill exercise for OA rats were based on the following protocols: speeds of 15.9, 19.2, and 26.8 m/min with 0°, 5°, and 10° inclination represented the OAL, OAM, and OAH groups, respectively. CG and OAG were the sedentary groups. Adaptive feeding and training from week 1 to week 2; experiment protocols were implemented from week 3 to week 6. After the last exercise session, rats were immediately sacrificed. **(B)** Weight change in rats during the study period. Differences between treadmill exercise (OAL, OAM, and OAH) and sedentary (CG and OAG) groups were significant. Conversely, differences among OAL, OAM, and OAH were not significant; ***p < 0.001; *n* = 8 rats for each group. Data were expressed as the mean ± SEM. **(C)** The optimum applied concentration of IL-1β in primary rat chondrocytes was evaluated using the CCK8 assay. The optimum applied concentration of IL-1β in primary rat chondrocytes was 10 ng/ml. The serum **(D)** and synovial fluid levels of irisin **(E)** in the treadmill exercise groups (OAL, OAM, and OAH) were higher than those in the sedentary groups (CG and OAG); those levels were especially higher in OAM. Values were expressed as the mean ± SEM. *p < 0.05 vs. CG; **p < 0.01 *vs*. CG; ***p < 0.001 *vs*. CG. ^#^ *p* < 0.05 *vs*. OAG; ^##^ *p* < 0.01 *vs*. OAG; ^###^ *p* < 0.001 *vs*. OAG. n = 8, means ±95% CI.

As shown in Figures 2D,E, the serum level and that of synovial fluid irisin were increased in all treadmill exercise groups (OAL, OAM, and OAH) compared with those in the sedentary groups (CG and OAG) as indicated by ELISA. Our results showed that treadmill exercise (especially OAM) could significantly (*p* < 0.001) increase the levels of irisin compared with that in the CG and OAG groups, and there was no statistically significant difference between OAH and OAL, OAM groups. As shown in Figure 2B, body weight differences between treadmill exercise (OAL, OAM, and OAH) and sedentary (CG and OAG) groups were significant (*p* < 0.001). Conversely, differences among OAL, OAM, and OAH were not significant (*p* = 0.874).

### 3.4 Histological Evaluation and Imaging Examination of Tibiofemoral Joints of Osteoarthritis Rats Under Different Treadmill Exercise Protocols

**FIGURE 3:**
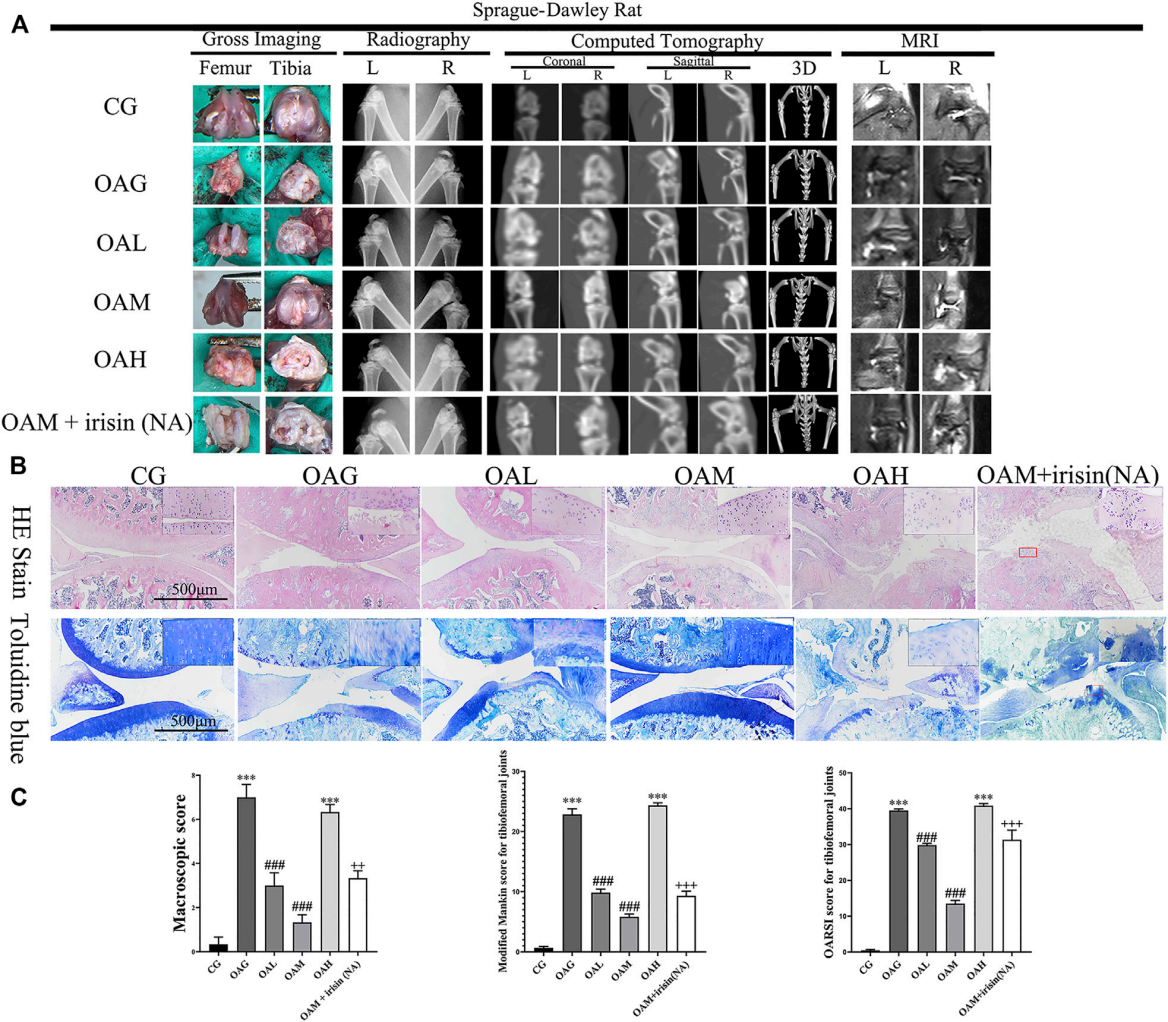
Histological evaluation and imaging examination of tibiofemoral joints of OA rats under different treadmill exercise protocols. OA model was confirmed using plain radiography, computed tomography (CT), and magnetic resonance imaging (MRI) **(A)**. Histological analysis was evaluated using hematoxylin and eosin (H&E) and toluidine blue **(B)** staining. We observed increased surface roughening, fibrillation, fissures, and erosions down to the subchondral bone in OAG and OAH compared with CG. A relatively smoother and complete articular cartilage surface was observed in OAM compared with OAG and OAH. However, the therapeutic effects of moderate-intensity treadmill exercise could be suppressed by irisin neutralizing antibodies. **(C)** Modified Mankin score for tibiofemoral joints. OARSI score for tibiofemoral joints. Data were expressed as the mean ± SEM. *p < 0.05 vs. CG; **p < 0.01 vs. CG; ***p < 0.001 vs. CG. #*p* < 0.05 vs. OAG; ##*p* < 0.01 vs. OAG; ###*p* < 0.001 vs. OAG. +*p* < 0.05 vs. OAM; ++*p* < 0.01 vs. OAM; +++*p* < 0.001 vs. OAM. *n* = 5, means ±95% CI.

We confirmed the occurrence of OA in our rat model using plain radiography, computed tomography (CT), and magnetic resonance imaging (MRI). We observed increased surface roughening, fibrillation, fissures, and erosions down to the subchondral bone in both the OAG and OAH groups compared with CG. We also noticed the occurrence of alleviations in the OAM and OAL groups compared with OAG and OAH (Figure 3A). We further evaluated our OA models using H&E and toluidine blue staining histological observations (Figure 3B). We found that the OAG and OAH groups showed cartilage damage and hypocellularity compared with the CG; OAH was worse than OAG. However, we observed that OAM displayed a relatively complete and smoother cartilage surface compared with that in OAG and OAH, indicating that OAM could ameliorate OA symptoms. Histological analysis based on the Modified Mankin and OARSI scores showed that the tibiofemoral joints in OAG and OAH exhibited reduced and damaged cartilage compared with that in CG. However, we found that compared with OAG and OAH, the articular cartilage injury was reversed in both OAL and OAM. In particular, the OAM group had the best therapeutic effect in tibiofemoral joints (Figure 3C). However, the therapeutic effects of moderate-intensity treadmill exercise could be suppressed by irisin neutralizing antibodies. The differences between OAM and OAM + irisin (NA) group are significant: macroscopic score (*p* < 0.01), Modified Mankin score (*p* < 0.001), OARSI score (*p* < 0.001).

### 3.5 Effects of Treadmill Exercise-Induced Irisin on the Expression of Inflammation-Related Proteins in Osteoarthritis Rat Models

**FIGURE 4:**
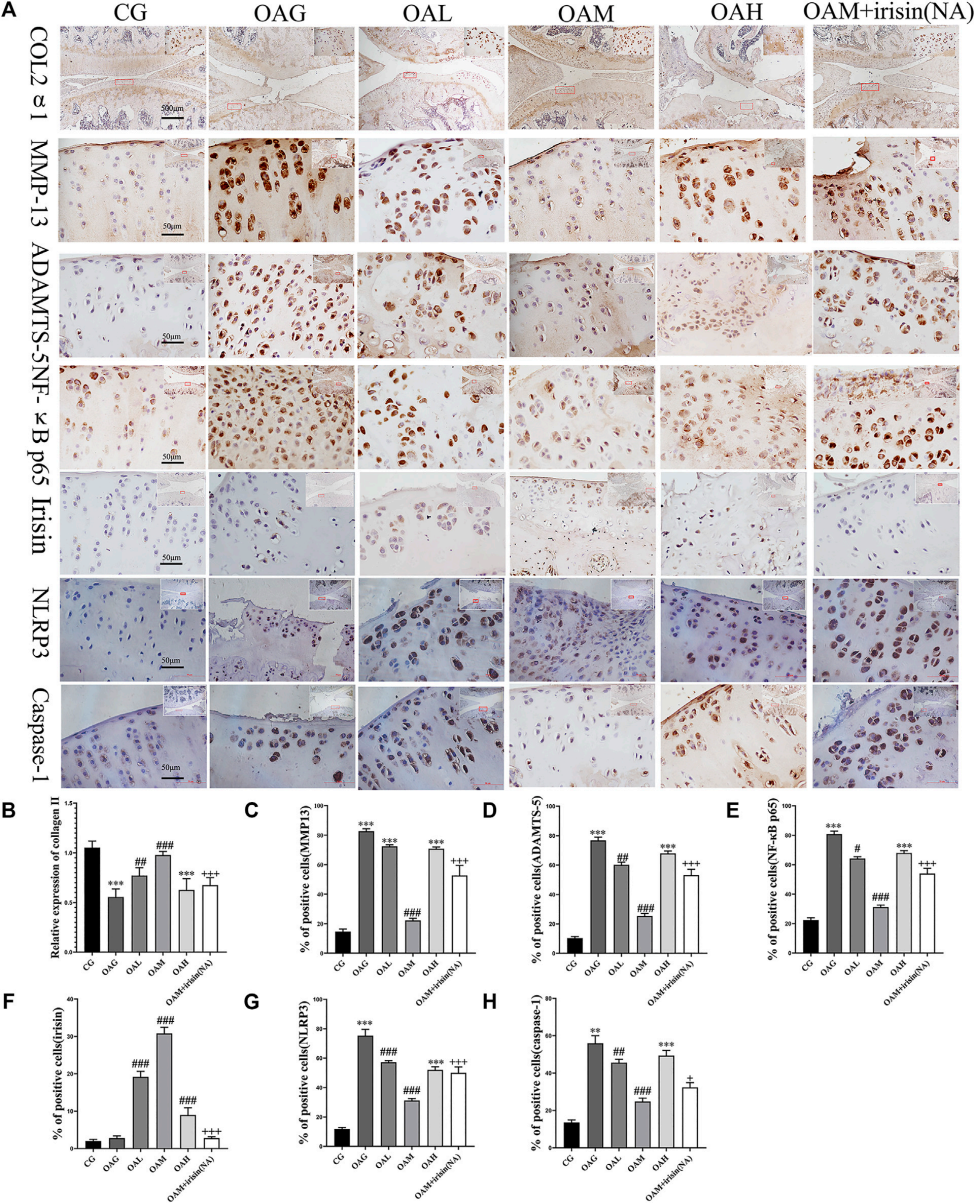
Immunohistochemical staining in OA rats subjected to different treadmill exercise protocols. **(A,B)** The expression of collagen II in CG, OAM, and OAL was higher than that in OAH and OAG. **(A,C)** The percentage of MMP-13 positive cells in CG and OAM was lower than that in OAH, OAG, and OAL. **(A,D)** The percentage of ADAMTS-5 positive cells in CG and OAM was lower than that in OAH and OAG. **(A,F)** The percentage of irisin positive cells in OAL, OAM, and OAH was higher than that in CG and OAG. **(A,G)** The percentage of NLRP3 positive cells in OAL, OAM, and OAH was lower than that in CG and OAG. **(A,H)** The percentage of caspase-1 positive cells in OAL, OAM, and OAH was lower than that in CG and OAG. However, the therapeutic effects of moderate-intensity treadmill exercise could be suppressed by irisin neutralizing antibodies. Data were expressed as the mean ± SEM. *p < 0.05 vs. CG; **p < 0.01 vs. CG; ***p < 0.001 vs. CG. ^#^ *p* < 0.05 vs. OAG; ^##^ *p* < 0.01 vs. OAG; ^###^ *p* < 0.001 vs. OAG. ^+^ *p* < 0.05 vs. OAM; ^++^ *p* < 0.01 vs. OAM; ^+++^ *p* < 0.001 vs. OAM. *n* = 5, means ±95% CI.

Our immunohistochemical staining revealed that the optical density of collagen II was higher in CG and OAM groups than in OAG and OAH groups. We further noticed that the percentage of positively stained MMP-13, ADAMTS-5, NLRP3, and caspase-1 cells was higher in OAG and OAH groups than in CG. Interestingly, this effect was recovered in OAM. We found that compared with OAG and OAH groups, the OAM group exhibited increased the expression of chondrocyte-specific collagen II (Figures 4A,B, but attenuation of MMP-13 and ADAMTS-5 in articular cartilage (Figures 4A,C,D). Our results also showed that the expression of irisin was decreased in the knee cartilage of the OA group but increased in all exercise groups, with particularly significant increases noted in the OAM group, and the upregulation of irisin could be blocked by irisin-neutralizing antibodies (Figures 4A,F). As shown in Figures 4A,G,H), the OAM group showed reversal of pyroptosis-related markers, such as NLRP3 and caspase-1. In addition, (Figures 4A–H), showed MMP-13, ADAMTS-5, NLRP3, and caspase-1 had significant differences between OAM and OAM + irisin (NA) groups, suggesting that the therapeutic effects are partially blocked by irisin neutralizing antibodies.

### 3.6 Effects of Irisin on IL-1β-Induced Chondrocyte Inflammation and Inflammatory-Related Protein/mRNA Expression

**FIGURE 5:**
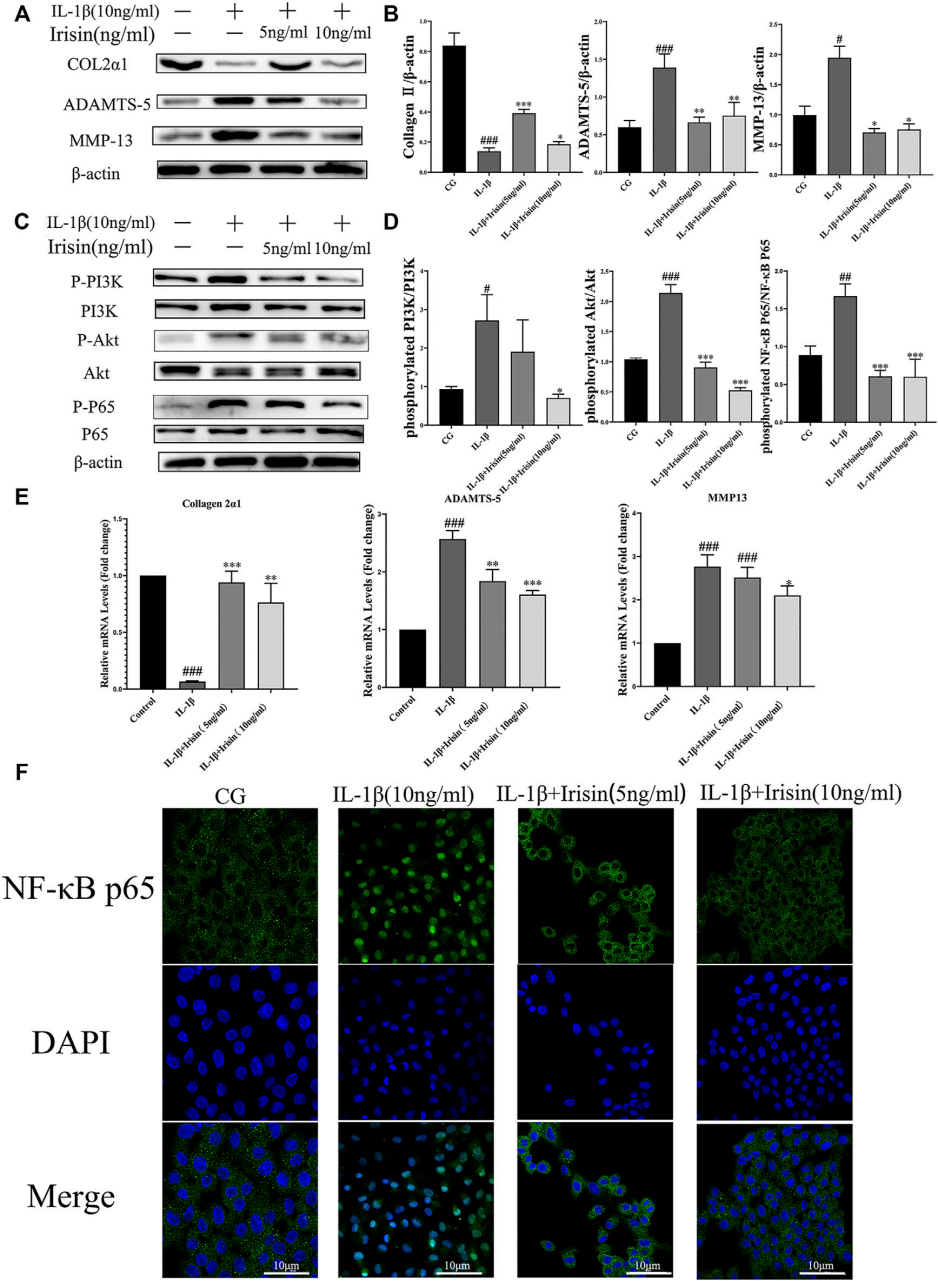
Expression of inflammation-related markers and immunofluorescence analysis of NF-κB p65 in chondrocytes. **(A–D)** The relative protein expressions were detected in the control (CG), IL-1β group, IL-1β + irisin (5 ng/ml), and IL-1β + irisin (10 ng/ml) groups. Chondrocytes were preincubated with different concentrations of irisin (0, 5, or 10 ng/ml) and treated with IL-1β (10 ng/ml) for 24 h. The levels of expression of ADAMTS-5 and MMP-13 were increased after treatment with IL-1β, whereas decreased after treatment with irisin. In addition, irisin-treated chondrocytes could partially recover the expression of chondrocyte-specific collagen II. The phosphorylation of PI3K, Akt, and NF-κB p65 was significantly suppressed in the irisin-treated group compared with that in the IL-1β group. **(E)** Relative mRNA expression of collagen II, ADAMTS-5, and MMP-13. The expression of IL-1β-induced inflammatory genes was inhibited by irisin in a dose-independent manner. **(F)** Effects of irisin on the nuclear translocation of NF-κB p65. Chondrocytes were immunostained using anti-NF-κB p65 rabbit antibody (green) and visualized under a confocal microcope. Cell nuclei were stained with DAPI (blue). Significant nuclear translocation of NF-κB p65 was detected in chondrocytes stimulated with IL-1β (10 ng/ml). In contrast, irisin intervention could reverse this trend. Data were expressed as the mean ± SEM, *p < 0.05 vs. IL-1β; **p < 0.01 vs. IL-1β; ***p < 0.001 vs. IL-1β. ^#^ *p* < 0.05 vs. CG; ^##^ *p* < 0.01 vs. CG; ^###^ *p* < 0.001 vs. CG. *n* = 3 for each group, means ±95% CI.

To further investigate the anti-inflammatory effects of exercise-induced irisin on OA chondrocytes, we performed western blot and qRT-PCR analyses. We first detected the relative protein expression in the CG, IL-1β, IL-1β + irisin (5 ng/ml), and IL-1β + irisin (10 ng/ml) groups (Figures 5A,B). We found that the levels of expression of ADAMTS-5 and MMP-13 proteins were upregulated by IL-1β but reversed after treatment with irisin. We further observed that the expression of chondrocyte-specific protein collagen II was recovered in irisin-treated chondrocytes. As shown in Figure 5E, IL-1β-treated chondrocytes showed upregulations of MMP-13 mRNA and ADAMTS-5 mRNA, whereas a downregulation of collagen II mRNA. However, we also noticed that the expression of IL-1β-induced inflammation mRNAs was inhibited with irisin treatment.

### 3.7 Effects of Irisin on the PI3K/Akt/NF-κB Signaling Pathway in IL-1β-Induced Osteoarthritis Chondrocytes

To explore the specific mechanism of irisin in OA chondrocytes, we evaluated the alterations in the PI3K/Akt/NF-κB signaling pathway in chondrocytes after treatment with IL-1β and irisin. In particular, we measured the levels of phosphorylation of PI3K, Akt, and NF-κB p65, which indicate the activation status of this cascade. As shown in Figures 5C,D, the levels of PI3K, Akt, and NF-κB phosphorylation were notably increased in the IL-1β group compared with those in CG, whereas they were suppressed in the irisin-treated group compared with those in the IL-1β group. We used immunofluorescence to investigate the effect of irisin on the nuclear translocation of NF-κB p65 in chondrocytes. We accordingly observed that NF-κB p65 was highly localized in the nuclei of IL-1β-treated chondrocytes compared with CG, indicating that IL-1β promoted the nuclear translocation of NF-κB p65. We further found that irisin pretreatment blocked the IL-1β-induced nuclear translocation of NF-κB p65 (Figure 5F). Collectively, irisin suppressed the IL-1β-induced chondrocyte inflammation by inhibiting the PI3K/Akt/NF-κB signaling pathway.

### 3.8 Effects of Irisin on the IL-1β-Induced Chondrocyte Pyroptosis

**FIGURE 6:**
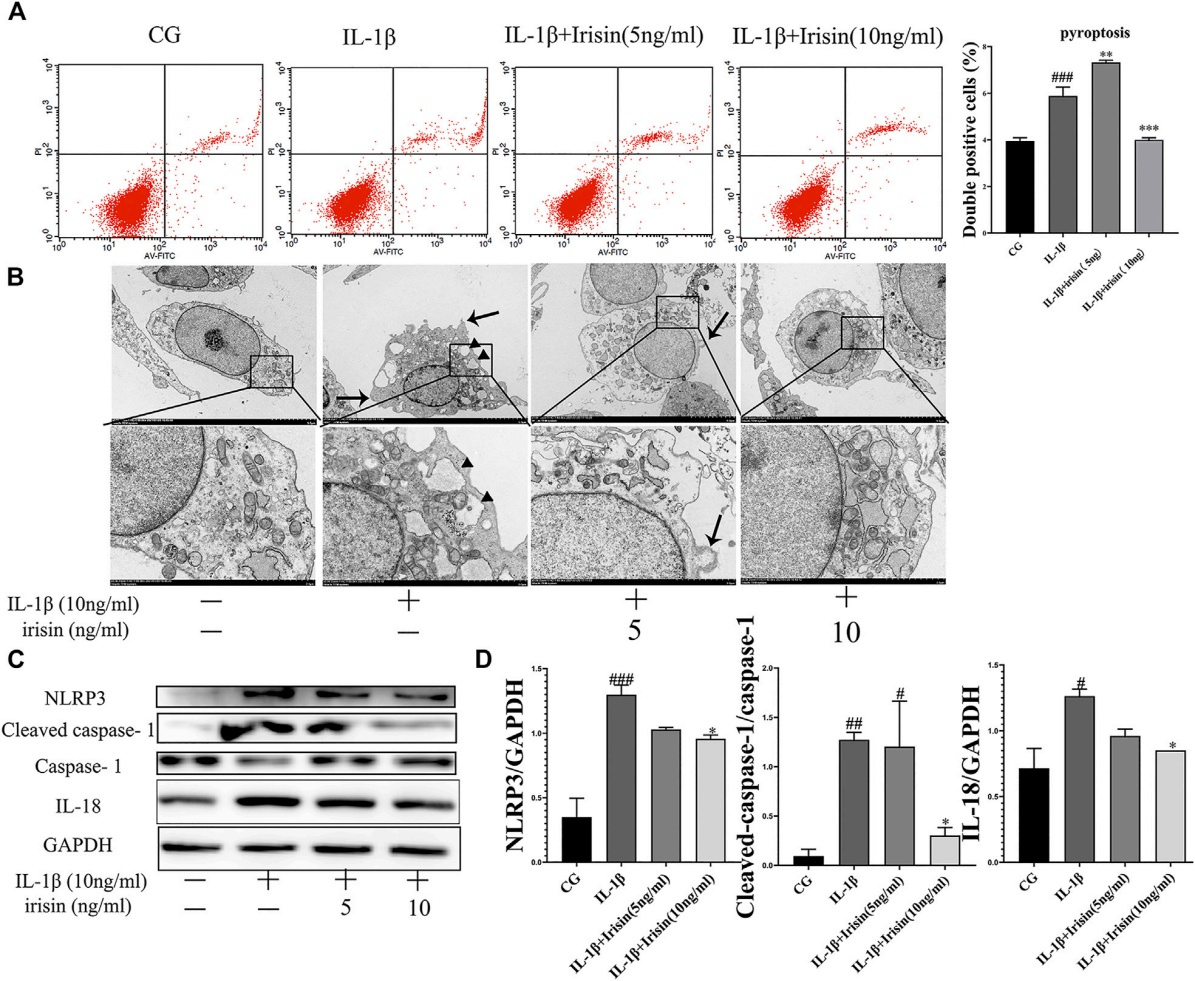
Irisin affects IL-1β-induced chondrocyte pyroptosis and inhibition of NLRP3/caspase-1 changes cellular morphology. **(A)** Effects of irisin on IL-1β-induced chondrocyte pyroptosis. **(B)** Representative TEM images of cellular morphological changes, such as cell swelling, cell membrane protrusion, and cell nucleus atrophy (scale bar: 5 μm). Black arrows indicate cell swelling, cell membrane protrusion; black triangle indicate organelle cavitation. **(C,D)** Representative western blots of the expression of pyroptosis-related proteins. GAPDH was used as control. Data were expressed as the mean ± SEM, *p < 0.05 vs. IL-1β; **p < 0.01 vs. IL-1β; ***p < 0.001 vs. IL-1β. ^#^ *p* < 0.05 vs. CG; ^##^ *p* < 0.01 vs. CG; ^###^ *p* < 0.001 vs. CG. *n* = 5 for each group, means ±95% CI.

We assessed the inflammation-induced chondrocyte death using flow cytometry. As shown in Figure 6A, irisin significantly (*p* < 0.001) decreased chondrocyte pyroptosis. We used transmission electron microscopy (TEM) to investigate the effect of irisin on inflammation-related pyroptosis. As shown in Figure 6B, the CG group exhibited the best cell morphology, with no characteristics of typical cell pyroptosis. More specifically, we observed that cells had a complete cell membrane with abundant pseudopods and protrudes visible around the membrane. In contrast, we noticed that chondrocytes in the IL-1β group exhibited a tendency for pyroptosis, including severe cytoplasmic edema, swelling of cell membrane, decreased overall electron density of cell matrix, and serious cavitation. However, we found that irisin inhibited the process of pyroptosis in irisin-treated chondrocytes, especially in the irisin (10 ng/ml)-treated group. We further observed that the NLRP3/caspase-1 pathway, which is key to pyroptosis was suppressed by irisin, as indicated in Figures 6C,D. Conclusively, irisin ameliorated the IL-1β-induced chondrocyte pyroptosis by inhibiting the NLRP3/caspase-1 pathway.

## 4 Discussion

In this study, we reported the therapeutic effects of treadmill exercise-induced irisin on OA both *in vivo* and *in vitro*. We found that treadmill exercise could significantly upregulate the serum and knee synovial fluid levels of irisin. In addition, exercise-induced irisin was shown to alleviate chondrocyte inflammation by inhibiting the PI3K/Akt/NF-κB signaling pathway, and ameliorate chondrocyte pyroptosis to treat OA.

In several previous studies, anaerobic and acute exercise were reported to increase the serum level of irisin; however, the exercise protocols were not specified (Schaalan et al., 2018; Tavassoli et al., 2019). In contrast, endurance and resistance exercise training were reported to reduce the circulating levels of irisin in some studies (Qiu et al., 2015). We hypothesized that these differences were influenced by the methods, intensities, or durations of exercise. However, no reliable model of exercise-induced irisin for the treatment of OA exists at present. Therefore, in the present study we established different intensities of treadmill exercise models to evaluate the level of irisin and its therapeutic effects on OA. Accordingly, we found that the circulating levels of irisin were significantly elevated after treadmill exercise, especially with moderate-intensity treadmill exercise. Our results not only established a reliable exercise protocol in rat model for exercise-induced irisin, but also confirmed the results of previous studies showing that moderate treadmill exercise alleviated OA cartilage damage in rat models (Martins et al., 2019; Bjerre-Bastos et al., 2021).

Interestingly, our immunohistochemical results found that exercise increased irisin-positive cell percentage in cartilage. This made us question whether irisin is locally produced in chondrocytes or contracting muscle secretes irisin to the blood, which then reaches the chondrocytes; alternatively, both mechanisms could be occurring simultaneously as well. In view of this finding, irisin neutralizing antibodies were added *in vivo* to the moderate-intensity exercise group, which were found to block local irisin expression and suppress the therapeutic effect observed in the OAM group. Based on the findings of this study and Boström et al. (2012), it is clear that irisin can be secreted by the muscle and released into the serum after exercise to participate in systemic circulation; notably, to our knowledge, no studies have shown that chondrocytes can express irisin. Second, as suggested by previous studies, chondrocytes are the only cells that form the articular cartilage and secrete the major components of the extracellular matrix (Krishnan and Grodzinsky, 2018; Varela-Eirin et al., 2018). Third, synovial fluid contributes to the unique functional properties of articular surfaces, provides nutrients to the cartilage, constitutes the microenvironment of chondrocytes, and modulates chondrocyte activity (Carlson et al., 2019). Therefore, we confirmed that the immunohistochemical staining of irisin (Figure 4A) was caused by the upregulation of irisin in the surrounding synovial fluid microenvironment, and concluded that muscle contractions lead to the production and secretion of irisin into the blood, which then reaches the chondrocytes *via* the synovial fluid. Our experimental results do not support the hypothesis that chondrocytes secrete irisin. In addition, the therapeutic effects of moderate-intensity exercise could also be partially blocked by irisin-neutralizing antibodies, suggesting that irisin is a mediator of exercise therapy for OA.

We found that high intensity treadmill exercise led to more severe damage of cartilage in OA, however, the irisin levels in OAH group were significantly higher than those in the sedentary groups (CG and OAG). We previously demonstrated that exercise could affect cartilage through mechanical stress, which had a dual effect on osteoarthritis. Adaptive mechanical stress can reduce the sensitivity of chondrocytes to inflammation. However, excessive mechanical stimulation leads to progressive damage, inhibits matrix synthesis, and stimulates the production of matrix degrading enzymes (Adams et al., 2009; Buckwalter et al., 2013; Yang et al., 2019; Yang et al., 2020). Secondly, exercise can also affect articular cartilage by altering the surrounding microenvironment, such as the synovial fluid (Akhbari et al., 2020). We found that treadmill exercise can significantly increase irisin concentration in synovial fluid, and we included *in vitro* experiments to determine that irisin can relieve inflammation and pyroptosis of chondrocytes. Thirdly, the effect of exercise on osteoarthritis is partially determined by mechanical stimulation and increased irisin concentrations. When excessive mechanical stimulation was applied to articular cartilage, the damage caused by high intensity exercise exceeded the treatment effect of elevated irisin concentrations. Therefore, high-intensity exercise aggravates the progression of OA. In this study, we focused on the balance between mechanical stimulation and elevated irisin concentrations for the treatment of osteoarthritis. We measured the effects of either low, moderate, or high intensity treadmill exercise with irisin levels to determine the maximum therapeutic effect. Our findings show that maintaining high circulating levels of irisin during adaptive exercise can achieve therapeutic effects in the treatment of OA. Thus, we concluded that adaptive mechanical stimulation protects against osteoarthritis through upregulating irisin.

Among the inflammatory cytokines that induce OA, IL-1β has been shown to play a critical role because it contributes to chondrocyte degradation by inducing the expression of MMP-13 and ADAMTS-5 during the pathogenesis of OA (Hu et al., 2019; Kapoor et al., 2011). Pyroptosis is a form of programmed inflammatory cell death, involving the activation of the NLRP3/caspase-1 which have been recognized as major markers of cell pyroptosis (Liu et al., 2019; Kesavardhana et al., 2020). We used a CCK8 assay to evaluate the optimum applied concentration of IL-1β (Figure 2C). As inflammation and inflammation induced-pyroptosis play important roles in the pathogenesis and development of OA, in-depth research on the inflammatory mechanism of OA would provide hope for the discovery and development of new disease-modifying therapies. In the present study, moderate-intensity treadmill exercise significantly increased the serum concentration of irisin and exerted the best therapeutic effects compared with other exercise intensities when evaluated using Modified Mankin and OARSI scores as well as following immunohistochemical analysis of MMP-13, ADAMTS-5, collagen II, and NF- κB p65. Therefore, we hypothesized that irisin is the key factor associated with exercise for the treatment of OA. Investigating the anti-inflammatory and antipyroptosis effects of irisin on chondrocytes is urgently needed to determine the specific mechanism.

In the present study, primary rat chondrocytes were isolated and cultured to evaluate the effects of irisin on OA *in vitro*. In the present study, primary rat chondrocytes were isolated and cultured to evaluate the effects of irisin on OA *in vitro*. Synovial fluid is formed by the serum in the articular capsule and released into the articular cavity. Synovial fluid contributes to the unique functional properties of articular surfaces, provides nutrients to the cartilage, constitutes the microenvironment of chondrocytes, and modulates chondrocyte activity (Akhbari et al., 2020). Therefore, we used synovial fluid concentration that approximated *in vivo* conditions, as determined by ELISA, to simulate cultured chondrocytes in our *in vitro* experiments. We found that irisin treatment significantly decreased the expression of IL-1β-induced inflammation-related genes and proteins, such as MMP-13 and ADAMTS-5, and inhibited the nuclear translocation of NF-κB p65. Irisin also enhanced the expression of chondrocyte-specific collagen II and inhibited the activity of nod-like receptor protein-3 (NLRP3)/caspase-1 to ameliorate pyroptosis in the chondrocytes.

**FIGURE 7:**
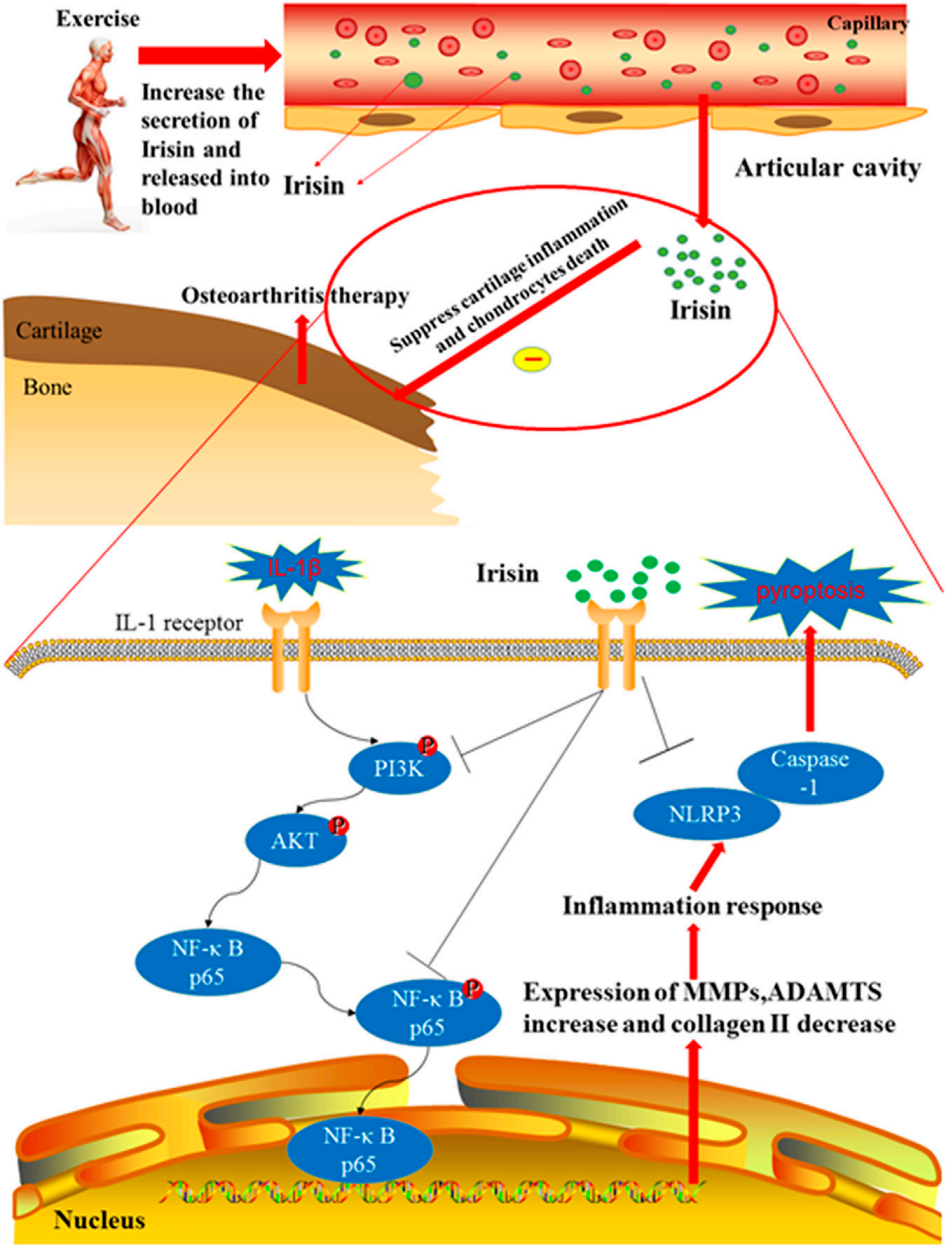
Therapeutic effects of irisin on OA during treadmill exercise via the inhibition of the PI3K/Akt/NF-κB signaling pathway and pyroptosis in chondrocytes. Irisin is secreted during appropriate treadmill exercise and then released into the blood, eventually reaching the knee articular cavity to play its role in inhibiting inflammation and pyroptosis. Exercise-induced irisin interaction with chondrocytes leads to the downregulation of inflammatory responses and suppression of cartilage matrix degradation, subsequently inhibiting pyroptosis in chondrocytes. In addition, irisin inhibits chondrocyte inflammation and inflammation-related pyroptosis in IL-1β-induced OA chondrocytes by inhibiting the PI3K/Akt/NF-κB signaling pathway.

Moderate-intensity treadmill exercise and irisin induced by moderate mechanical stimulation have been proposed to ameliorate the progression of OA. Our *in vitro* experiments demonstrate that irisin suppressed IL-1β-induced chondrocyte inflammation, reduced pyroptosis, and blocked activation of the PI3K/Akt/NF-κB cascade in the cultured OA chondrocytes. Taken together, these findings indicate that moderate mechanical stimulation protects against chondrocyte pyroptosis through irisin-induced suppression of PI3K/Akt/NF-κB signal pathway in osteoarthritis. This proposed mechanism is illustrated in Figure 7. Our results also confirmed those of a previous study by Vadala et al. (2020), who suggested that irisin could reduce the expression of type X collagen, as well as IL-1, IL-6, MMP-1, MMP-13, and inducible nitric oxide synthase (iNOS) through inactivating the p38 MAPK, Akt, JNK, and NFκB signaling pathways. Li et al. (2021) reported that irisin is involved in chondrogenesis and the pathogenesis of OA, and abnormal changes in the expression of irisin in OA cartilage suggest that irisin is a promising therapeutic target. To further validate our findings, we established an exercise protocol to measure exercise-induced irisin levels and investigate the role of irisin in exercise therapy for treating OA. Based on the differential expression of exercise-related genes from our bioinformatics analysis, we demonstrated that irisin can indeed suppress activation of the PI3K/Akt/NF-κB cascade. We also confirmed that irisin can inhibit inflammation-induced chondrocyte pyroptosis to ameliorate OA.

Our study has several limitations. First, considering the significant differences in the biomechanics of human and rat knee joints, the protocol for exercise-induced irisin used in this study cannot be directly extrapolated to human studies. Our next step is to rework this protocol for better applicability to human studies. Second, we did not account for other signaling pathways that are known to be involved in NF-kB and inflammasome activation and have previously been reported to be inhibited by irisin. In subsequent experiments, we will take into consideration other signaling pathways. Third, in this study, we focused on the balance between mechanical stimulation and elevated irisin concentrations for the treatment of OA. In future studies, we will directly study the *in vivo* effect of irisin in the absence of exercise.

## 5 Conclusion

In summary, moderate mechanical stimulation protects against osteoarthritis through irisin, but aggravated OA during high-intensity exercise *in vivo*. Furthermore, exercise-induced irisin suppressed IL-1β-induced inflammation in rat OA chondrocytes via the inhibition of the PI3K/Akt/NF-κB signaling pathway and activity of NLRP3/caspase-1, ameliorating pyroptosis in chondrocytes. Collectively, moderate intensity exercise might be considered a novel therapeutic agent for OA, and irisin might be a mediator of the therapeutic effect. Our results not only provided an important reference for OA therapeutics but could also be used to guide exercise therapy, minimize the side-effects of sports, reduce OA incidence, and help determine the clinical treatment of OA.

## Data Availability

The data presented in the study can be accessed via the link: https://doi.org/10.6084/m9.figshare.19315841.v1.
