# Supplementary material for: Mechanical Stimulation Protects Against Chondrocyte Pyroptosis Through Irisin-Induced Suppression of PI3K/Akt/NF-κB Signal Pathway in Osteoarthritis
Source: Front Cell Dev Biol. 2022 Mar 9;10:797855. doi: 10.3389/fcell.2022.797855 (PMC8959944; doi:10.3389/fcell.2022.797855)
Supplement: Supplementary file 1 [file DataSheet1.DOCX]

Supplementary Material

# Supplementary Materials


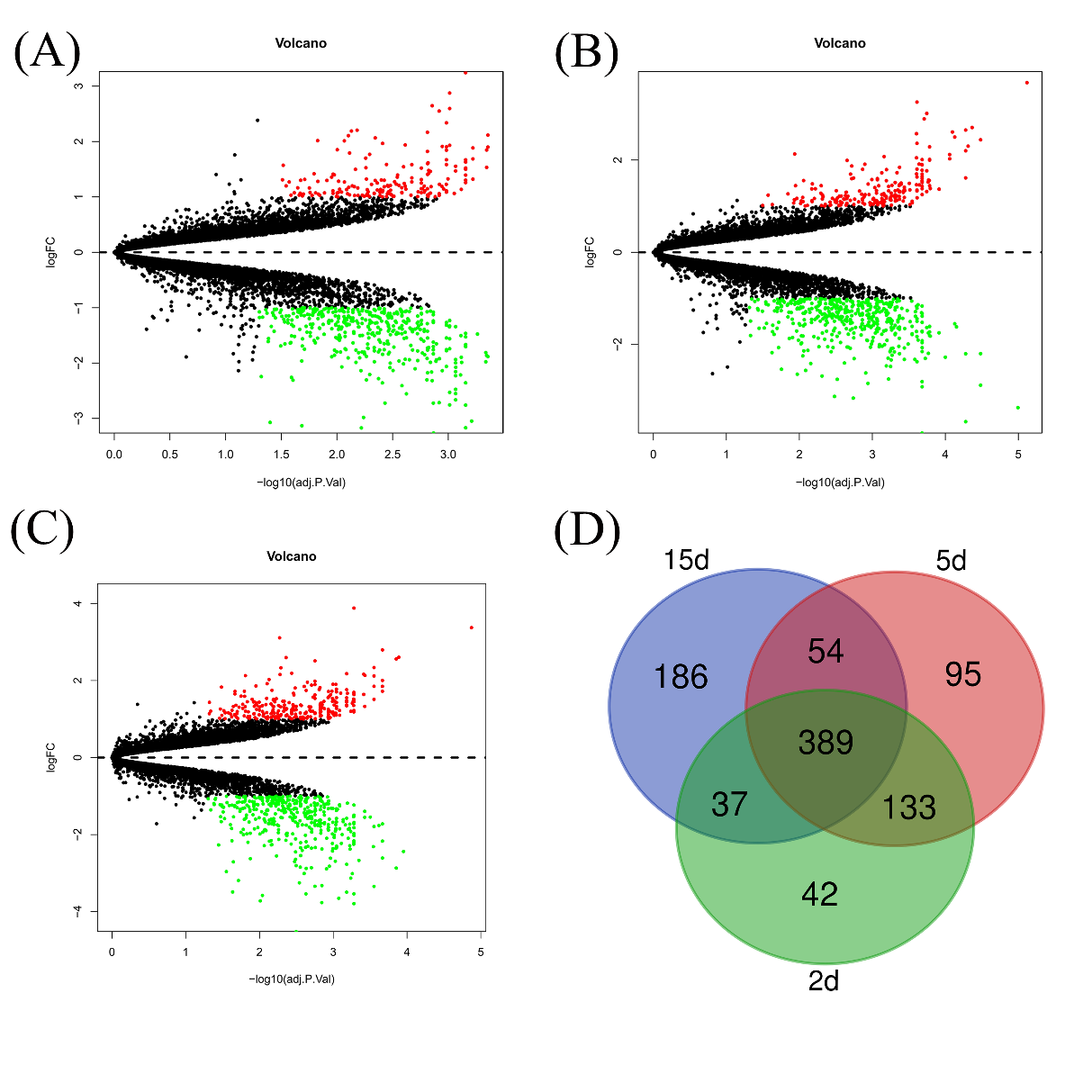


**Figure S1. Volcano plots and Venn diagram of relative genes.** The black dots represent the expression of genes which have no differences between the cartilage of control group and 2-, 5-, or 15-day exercise groups. The red dots represent the upregulated genes and the green dots represent downregulated genes (A-C). The Venn diagram of these exercise-related differential genes (D).

**
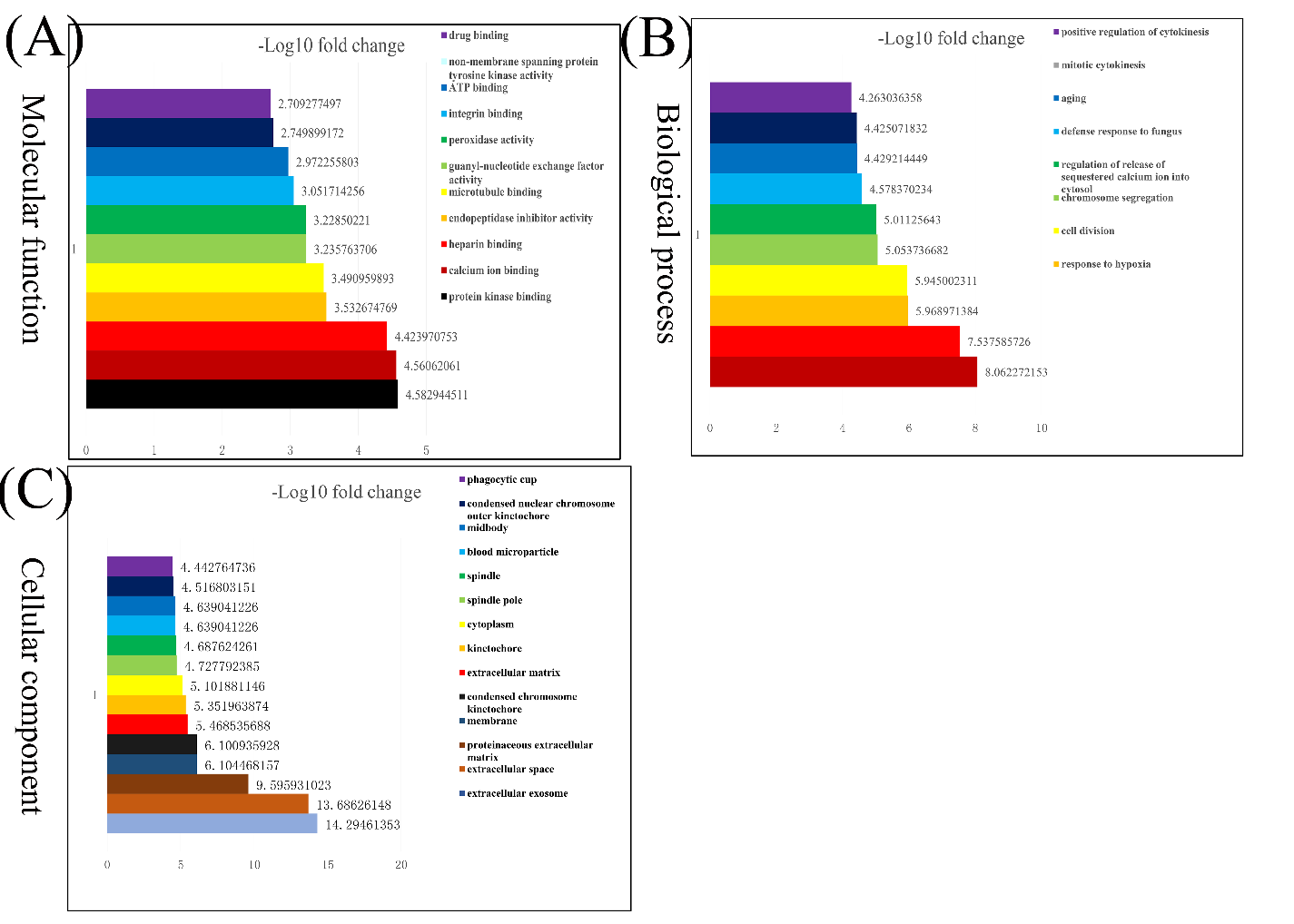
**

**Figure S2.** **Gene ontology (GO) functional annotation analysis.** These three GO functional annotation analysis were adopted on the exercise-related differentially expressed genes. Molecular function (A), biological process (B), cellular component (C).


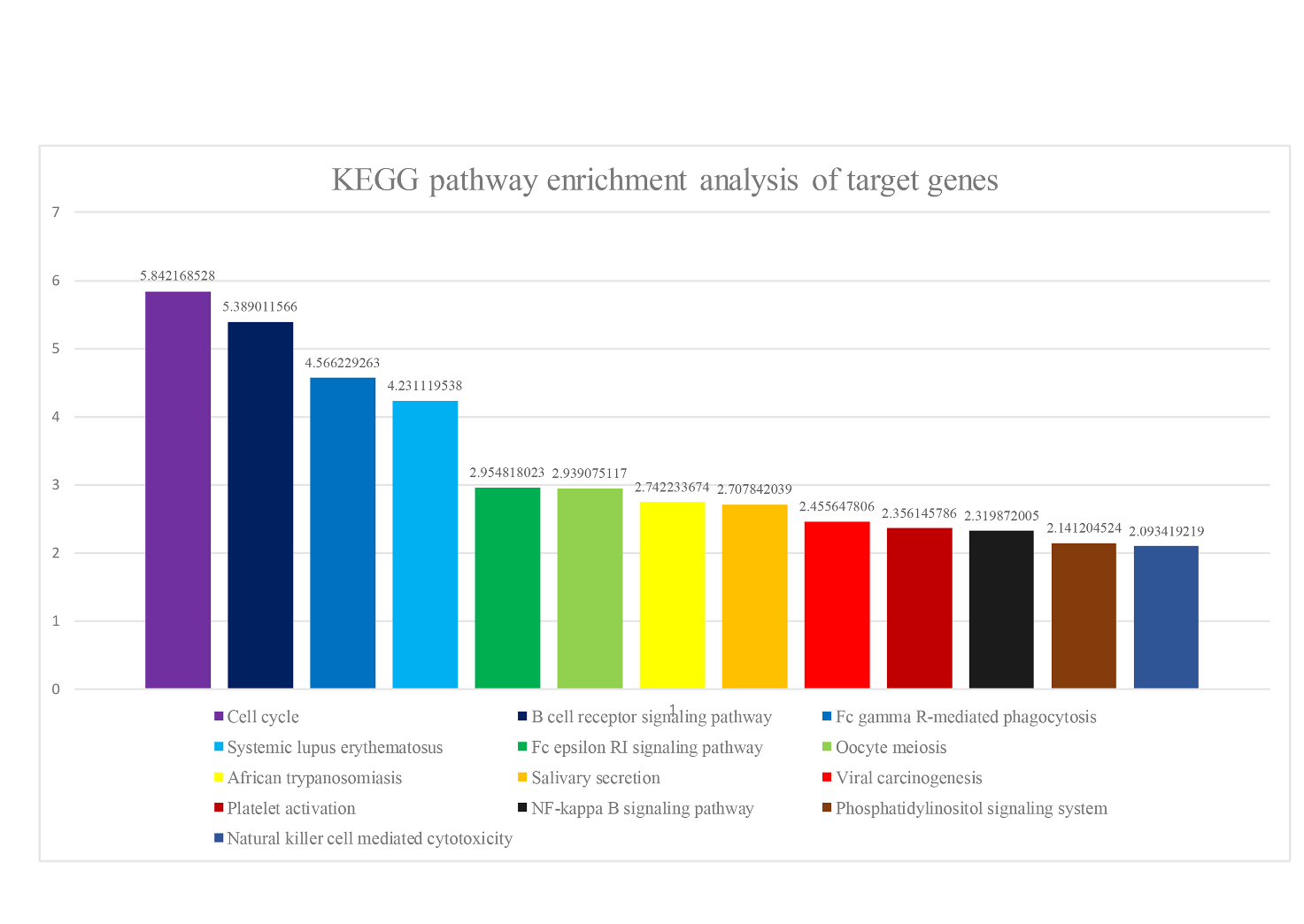


**Figure S3.** **Kyoto Encyclopedia of Genes and Genomes (KEGG) pathway enrichment analysis** **of the exercise-related differentially expressed genes**.
